# Fiberoptic endoscopic evaluation of swallowing and the Brazilian version of the Eating Assessment Tool-10 in resistant hypertensive patients with obstructive sleep apnea

**DOI:** 10.1016/j.bjorl.2022.01.006

**Published:** 2022-02-10

**Authors:** Mariana Pinheiro Brendim, Carla Rocha Muniz, Thalýta Georgia Vieira Borges, Flávia Rodrigues Ferreira, Elizabeth Silaid Muxfeldt

**Affiliations:** a Universidade Federal do Rio de Janeiro, Faculdade de Medicina, Programa de Pós-Graduação em Clínica Médica, Departamento de Clínica Médica, Rio de Janeiro, RJ, Brazil; b Universidade Federal do Rio de Janeiro, Faculdade de Medicina, Departamento de Fonoaudiologia, Rio de Janeiro, RJ, Brazil

**Keywords:** Sleep apnea, Obstructive, Deglutition disorders, Fiberoptic endoscopic evaluation of swallowing, ROC curve, Screening

## Abstract

**Objective:**

The aim of this study was to describe the prevalence and characteristics of OD through Fiberoptic Endoscopic Evaluation of Swallowing (FEES) and the Eating Assessment Tool-10 (EAT-10) in hypertensive patients with OSA, as well as to describe the sensitivity of EAT-10 for the detection of OD in this population.

**Methods:**

This study included a convenience sample in which 85 resistant hypertensive patients diagnosed with OSA in an university hospital participated. Participants were subjected to the EAT-10 (index test) and FEES (reference standard).

**Results:**

The median EAT-10 score was 2 (0–5.5). According to the FEES, 27 participants did not have dysphagia, 42 had mild dysphagia and 16 had mild to moderate dysphagia. The sensitivity of the EAT-10 was 70.7% (95% CI: 57.3–81.9) at a cutoff score ≥1, with a discriminatory power of 67.4% (*p* = 0.005). The most prevalent symptom in this population was “food stuck in the throat”, while the most prevalent signs were delayed initiation of the pharyngeal phase of swallowing, premature bolus spillage and pharyngeal residue.

**Conclusion:**

In our study, the cutoff score for the EAT-10 for screening for OD in this population was ≥ 1. In conclusion, this population presented a high prevalence of dysphagia detected in FEES and its severity is associated with higher EAT-10 scores.

## Highlights

• Resistant hypertensive with OSA have a high prevalence of deglutition disorders.
• Changes in the oral phase of swallowing are inferred by FEES in these subjects.
• Changes in the pharyngeal phase of swallowing are found by FEES in these subjects.
• The greater severity of dysphagia is associated with higher EAT-10 scores.
• The cutoff score for EAT-10 for screening of dysphagia is ≥1 in these subjects.

## Introduction

Obstructive Sleep Apnea (OSA) and resistant hypertension are conditions that have been strongly associated,^1^ with the first characterized by the repetitive collapse of the upper airways during sleep,^2^ causing ≥5 obstructive respiratory events per hour of sleep associated with symptoms of sleep disturbance or ≥15 per hour of sleep even without symptoms.^2^ The second is characterized by having blood pressure above goal despite the use of three drug classes, commonly including a diuretic, a inhibitor of the renin-angiotensin-aldosterone system and a calcium channel blocker, or by blood pressure controlled by use four or more drugs.^3^

Individuals with OSA have pharyngeal sensory and muscle changes,^4^, ^5^, ^6^, ^7^, ^8^, ^9^ possibly due to repetitive vibratory trauma caused by snoring,^6^ pharyngeal microcirculation disorder,^10^ inflammatory changes,^10^ and hypoxia and hypercapnia.^9^ It is believed that these changes may cause swallowing dysfunction,^11^ thus explaining the signs of Oropharyngeal Dysphagia (OD) described in the literature in this population,^11^, ^12^, ^13^, ^14^, ^15^, ^16^, ^17^, ^18^, ^19^ such as premature bolus spillage,^12^, ^13^, ^14^, ^15^, ^16^, ^17^, ^18^, ^19^ pharyngeal residue,^12^, ^13^, ^14^, ^15^, ^16^, ^19^ penetration^12^, ^14^, ^16^, ^18^^,^^19^ and, less frequently, aspiration.^14^ It is noteworthy that not all changes in swallowing function are dysphagia, however changes with an impact on the safety and efficacy of swallowing are considered to be dysphagia.^11^ Therefore, clinical and instrumental exams, such as videofluoroscopic swallowing study and Fiberoptic Endoscopic Evaluation of Swallowing (FEES), added to the description of symptoms and clinical history can determine the diagnosis of OD.

However, few studies have investigated the location of the residue, and none have investigated when penetration-aspiration occurs, which are considered important aspects for understanding swallowing disorders^20^ and consequently therapeutic planning for swallowing rehabilitation. In addition, the manifestations of OD in this population are subclinical,^13^, ^14^, ^17^, ^19^^,^^21^ and it is essential that clinicians include questions about swallowing when collecting the history of these individuals^14^ having an assessment instrument for OD symptoms appropriate to detect this manifestation in individuals with OSA.

The Eating Assessment Tool (EAT-10) consists of a self-assessment questionnaire for dysphagia symptoms, which allows the risk of dysphagia to be identified^22^ and has been cross-culturally adapted to several languages, including Brazilian Portuguese.^23^ It is considered simple, fast and easy to apply and has the possibility of being used by several types of health professionals and in any patient with suspected dysphagia who is able to answer it.^22^, ^23^

Thus, the objective of this article was to describe the prevalence and characteristics of OD through FEES and the Brazilian version of the EAT-10 in resistant hypertensive patients with OSA. In addition, this study aimed to assess whether there was a relationship between EAT-10 scores and the swallowing changes evidenced by FEES, as well as to describe the sensitivity of the EAT-10 for the detection of OD in this population.

## Methods

A study was approved by the Research Ethics Committee (number 1.348.512). All study participants signed an informed consent form.

### Participants

A convenience series of resistant hypertensive patients with OSA from the Hypertension Program of tertiary-care University Hospital Clementino Fraga Filho, Rio de Janeiro, Brazil, who were aged ≥ 18 years and diagnosed with OSA through full-night polysomnography at the sleep laboratory of this same hospital were recruited. Individuals > 65 years, individuals with neurological disease, head and neck cancer, chronic obstructive pulmonary disease, vocal fold paralysis, tracheostomy, and cognitive deficits, and individuals who did not consent to participation in the research were excluded. In addition, individuals hospitalized for kidney transplantation during the study period were excluded.

During polysomnography performed with the BrainNet BNT (EMSA, Brazil) and supervised by a clinician, electroencephalogram, electrooculogram, submentonian electromyogram, nasal airflow, oximetry, respiratory effort, electrocardiogram, and anterior tibial electromyogram were recorded. The examination report was prepared by a clinician, who was a specialist certified in Sleep Medicine, who was unaware of the patients' clinical data.

Apnea and hypopnea were defined as ≥ 90% and ≥ 30% reductions, respectively, in airflow for at least 10 s associated with ≥ 4% oxyhemoglobin desaturation.^24^ Obstructive and central apneas were diagnosed according to the presence or absence of respiratory effort.^24^ The Apnea-Hypopnea Index (AHI) was calculated as the number of apnea-hypopnea events per hour of sleep. The severity of OSA was defined as mild (5 ≤ AHI < 15), moderate (15 ≤ AHI ≤ 30) or severe (AHI > 30).^2^

### Study design

This was a cross-sectional study whose data collection was planned before the index test (EAT-10) and the reference standard (FEES). The study was carried out at the otorhinolaryngology service of University Hospital Clementino Fraga Filho between February 2016 and July 2018. All participants completed a questionnaire on demographic and clinical characteristics followed, on the same day, by the application of the EAT-10 and FEES.

### Index test

The application of the EAT-10 was performed by a speech therapist blinded to the participants' clinical data. Although the EAT-10 is self-applied, due to the socioeducational level of the participants, we opted for the reading of the items by a speech therapist, who transcribed the answers provided by the participants. The EAT-10 consists of 10 items, covering functional (Items 3–5), emotional (Items 2, 7 and 10) and physical (Items 1, 6, 8 and 9) domains, with responses for each item ranging from 0 to 4 points, for a total score between 0 and 40 points. The Brazilian version, like the original, has a cutoff score ≥3 points for the risk of dysphagia.^23^

### Reference standard

Immediately after completing the EAT-10, the participants underwent FEES. The evaluation was performed using ENT-30PIII Machida nasofibroendoscopy equipment by an otolaryngologist and a speech therapist who were blinded to the clinical data and the EAT-10 results of the participants. Boluses of 5 mL, 10 mL and 15 mL of liquid, nectar, honey, and pudding consistencies were evaluated, in addition to ½ cookie (Club Social®). Two trials were considered for each volume and consistency. The Nestlé ThickenUp® Clear thickener diluted in water, in an amount recommended by the manufacturer, was used to control the consistencies. A third supply of 5 mL of liquid was used to assess velopharyngeal closure during swallowing, when the endoscope was positioned in the nasopharynx. All foods were stained with blue food coloring. The FEES was performed with the patient seated at 90°, without using topical anesthetic, following the endoscopic positioning techniques recommended in the literature^25^ and according to the protocol proposed by Langmore.^20^

The exams were subsequently evaluated, independently and blindly, in real time and frame-by-frame by two speech therapists experienced in dysphagia. In situations of disagreement, an ENT specialist with FEES expertise was consulted. The analyzed parameters were as follows:

Premature bolus spillage: identified as parts of the bolus falling piecemeal into the pharynx before the swallow^25^ (i.e., before the ejection movement of the base of the tongue).

Delayed initiation of the pharyngeal phase of swallowing: (0) no delay; (1) head of bolus in valleculae before initiation of pharyngeal phase of swallowing; (2) head of bolus in pyriform sinuses or lower before initiation of pharyngeal phase of swallowing.^26^ A score > 1 was considered delayed initiation of the pharyngeal phase of swallowing since healthy and asymptomatic individuals frequently exhibit boluses in the vallecula before initiation of the pharyngeal phase of swallowing.^27^

Piecemeal deglutition: defined by dividing a bolus into two or more successive swallows.^14^

Delayed posterior leakage: included in the analysis since the evaluators perceived signal characterized by posterior leakage of the bolus after the physiologically completed swallowing.^28^

Penetration-aspiration: the level of penetration-aspiration was determined by the penetration-aspiration scale.^29^ In the case of abnormal penetration-aspiration scores (>2), whether penetration-aspiration occurred before, during or after swallowing was also evaluated.^25^

Pharyngeal residue: the degree of residue was determined using the Yale pharyngeal residue severity rating scale.^30^ In cases with pharyngeal residue, the location of residue was determined.

Velopharyngeal insufficiency: presence or absence of nasal reflux of the bolus.

Dysphagia severity: the degree of OD was determined by the dysphagia severity scale.^31^

The choice of FEES as a reference standard was because this exam, along with videofluoroscopy, are the two most widely used methods for assessing OD, and FEES has greater sensitivity for identifying aspiration, penetration and residue compared to videofluoroscopy.^32^

### Statistical analysis

Statistical analysis was performed using SPSS 19.0 software. Agreement between evaluators was analyzed using the Kappa index (κ). The normality of the data was checked by histograms and quantile–quantile plots, while the homogeneity of variance was checked by the Levene test. Independent samples *t*-test or ANOVA was used in cases where the assumptions of normality and homogeneity of variance were satisfied, and the Mann–Whitney test or Kruskal–Wallis test was used when these assumptions were not satisfied. The Receiver Operating Characteristic (ROC) curve analyses as well as the sensitivity, specificity, accuracy, predictive values, and likelihood ratios of the EAT-10 were analyzed using MedCalc software, version 19.0.4. The level of statistical significance adopted was 5%.

## Results

**Fig. 1:**
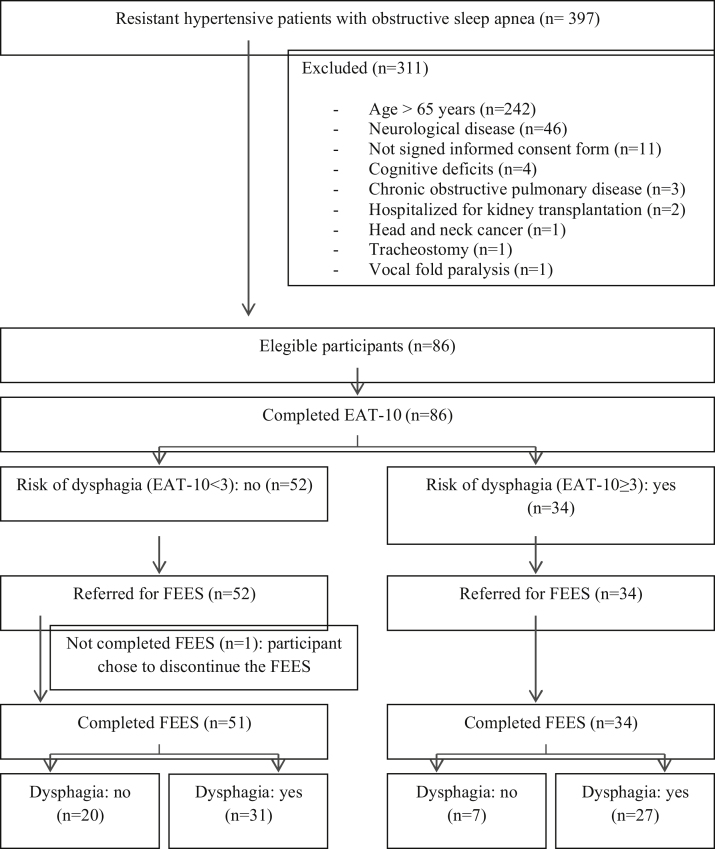
Flow diagram of study participants.

Of the 397 hypertensive individuals resistant to OSA, 86 were eligible, but one participant chose to discontinue the FEES (Fig. 1).

The final sample consisted of 85 participants, aged between 34 and 65 years and an AHI between 6 and 100. The prevalence of OD diagnosed by FEES was 68.2%. The baseline characteristics are described in Table 1, with no significant differences between the groups with and without OD (Table 1).

**Table 1:** Baseline characteristics of participants grouped according to presence or absence of oropharyngeal dysphagia.

Characteristics	Total	Oropharyngeal dysphagia		*p*-Value
	n = 85	No (n = 27)	Yes (n = 58)	
Sex, females, n (%)	63 (74.1)	18 (66.7)	45 (77.6)	0.285^a^
Age (years), median (IQR)	60 (55–63)	58 (54–62)	61 (55.75–63.25)	0.080^b^
Smoking, n (%)	9 (10.6)	4 (14.8)	5 (8.6)	0.305^c^
Weight (kg), median (IQR)	84 (75.5–103.5)	81 (77–109)	86 (73–103.25)	0.959^b^
Height (m), mean ± SD	1.62 ± 0.01	1.63 ± 0.1	1.61 ± 0.1	0.447^d^
BMI (kg/m^2^), median (IQR)	33.7 (28.7–37)	32.2 (28.7–36.2)	34.2 (28.7–37.2)	0.549^b^
NC (cm), mean ± SD	41.4 ± 4.6	42.3 ± 3.99	41 ± 4.83	0.204^d^
AHI, median (IQR)	22 (10–38)	22 (8–38)	23 (12–36.25)	0.558^b^
CPAP, n (%)	19 (22.4)	5 (18.5)	14 (24.1)	0.563^a^
Severity of OSA, n (%)				
Mild	30 (35.3)	12 (44.4)	18 (31)	0.366^a^
Moderate	23 (27.1)	5 (18.5)	18 (31)	
Severe	32 (37.6)	10 (37)	22 (37.9)	

BMI, body mass index; NC, neck circumference; AHI, apnea-hypopnea index; OSA, obstructive sleep apnea; IQR, interquartil range; SD, standard deviation.

a Pearson’s Chi-Square test.

b Mann–Whitney test.

c Fisher’s exact test.

d Independent samples *t*-test.

**Fig. 2:**
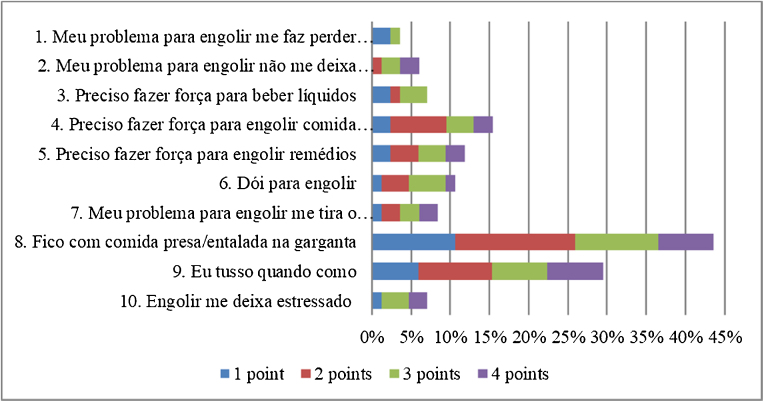
Distribution of the EAT-10 scores.

Although no participant had a spontaneous complaint of dysphagia, 51 (60%) scored on at least one item of the EAT-10. The total EAT-10 score varied between 0 and 29, with a median of 2 (0–5.5). The median for each item was equal to 0 (0‒0), with the exception of items 8 and 9, whose medians were equal to 0 (0–2) and 0 (0–1), respectively. The distribution of the EAT-10 scores is shown in Fig. 2. The domain most affected was the physical domain, with a median of 1 (0–3), followed by the functional and emotional domains, both equal to 0 (0‒0) (Fig. 2).

The agreement between the evaluators in the FEES was high, and no adverse effects were observed during the procedure. A total of 67.1% of the participants had premature bolus spillage (κ = 0.805); 70.6%, delayed onset of the pharyngeal phase of swallowing (κ = 0.916); 47.1%, piecemeal deglutition (κ = 0.929); 47.1%, delayed posterior leakage (κ = 0.906); 34.1%, penetration-aspiration (κ = 1.000); 61.2%, pharyngeal residue (κ = 0.950); and 0%, nasal reflux (κ = 1.000).

Regarding delayed onset of pharyngeal triggering, 1.2% of the participants exhibited no delay, 28.2% exhibited head of bolus in valleculae, and 70.6% exhibited head of bolus in pyriform sinuses or lower before initiation of the pharyngeal phase of swallowing (κ = 0.917).

Regarding the penetration-aspiration scale, 24.7% of the participants exhibited 1 point; 41.2%, 2 points; 10.6%, 3 points; 16.5%, 4 points; 5.9%, 5 points; and 1.2%, 6 points (κ = 0.984). In cases of penetration-aspiration, 35.2% occurred before swallowing, 58.1% during swallowing and 6.7% after swallowing (κ = 0.915).

Regarding the level of pharyngeal residue, 24.7% of the participants exhibited none; 14.1%, trace; 38.8%, mild; 18.8%, moderate; and 3.5%, severe (κ = 0.919). Regarding the location of the pharyngeal residue, 1.2% occurred on the base of the tongue; 77.7%, in the vallecula; 3.7%, in the pyriform sinus; 2%, in the retrocricoid area; and 15.4%, in both the vallecula and pyriform sinus (κ = 0.875).

In reference to the severity of dysphagia, 5.9% of the participants had normal swallowing; 25.9%, functional swallowing; 49.4%, mild dysphagia; and 18.8%, mild to moderate dysphagia (κ = 0.964).

The medians of EAT-10 scores in the groups with normal swallowing, functional swallowing, mild dysphagia and mild to moderate dysphagia were 0 (0‒0), 0 (0–3), 2 (0–6) and 2 (0.25–10.75), respectively. There was a statistically significant difference between the EAT-10 scores and the severity of OD (Kruskal–Wallis test: *p* = 0.015). The relationship between the scores for each EAT-10 item and the signs of swallowing dysfunction is shown in Table 2. There was a relationship between the scores on Item 8 and residue, delayed posterior leakage and OD. There was also a relationship between the scores on Item 5 and delayed posterior leakage, between the scores on Item 7 and premature bolus spillage, and between the EAT-10 total scores and delayed posterior leakage and OD. In addition, there was a relationship between the scores on Items 2 and 9 and penetration-aspiration. There were no relationships between the EAT-10 scores and the variables delayed initiation of the pharyngeal phase of swallowing and piecemeal deglutition (Table 2).

**Table 2:** Relationship between the scores for each EAT-10 item and the signs of swallowing dysfunction.

Signs		Item 1	Item 2	Item 3	Item 4	Item 5	Item 6	Item 7	Item 8	Item 9	Item 10	EAT-10
Premature spillage	No	0 (0–0)	0 (0–0)	0 (0–0)	0 (0–0)	0 (0–0)	0 (0–0)	0 (0–0)	0 (0–1)	0 (0–2)	0 (0–0)	1 (0–6.75)
	Yes	0 (0–0)	0 (0–0)	0 (0–0)	0 (0–0)	0 (0–0)	0 (0–0)	0 (0–0)^a^	1 (0–2)	0 (0–0)	0 (0–0)	2 (0–4)
Delayed onset of the PR	No	0 (0–0)	0 (0–0)	0 (0–0)	0 (0–0)	0 (0–0)	0 (0–0)	0 (0–0)	0 (0–2)	0 (0–1)	0 (0–0)	0 (0–4)
	Yes	0 (0–0)	0 (0–0)	0 (0–0)	0 (0–0)	0 (0–0)	0 (0–0)	0 (0–0)	0 (0–2)	0 (0–1.75)	0 (0–0)	2 (0–6)
Piecemeal deglutition	No	0 (0–0)	0 (0–0)	0 (0–0)	0 (0–0)	0 (0–0)	0 (0–0)	0 (0–0)	0 (0–2)	0 (0–1)	0 (0–0)	1 (0–3.5)
	Yes	0 (0–0)	0 (0–0)	0 (0–0)	0 (0–0)	0 (0–0)	0 (0–0)	0 (0–0)	0 (0–2)	0 (0–1.75)	0 (0–0)	2 (0–6)
Delayed posterior leakage	No	0 (0–0)	0 (0–0)	0 (0–0)	0 (0–0)	0 (0–0)	0 (0–0)	0 (0–0)	0 (0–1)	0 (0–1)	0 (0–0)	1 (0–3)
	Yes	0 (0–0)	0 (0–0)	0 (0–0)	0 (0–0)	0 (0–0)^a^	0 (0–0)	0 (0–0)	1 (0–3)^a^	0 (0–2)	0 (0–0)	3 (0–6)^a^
Penetration- aspiration	No	0 (0–0)	0 (0–0)	0 (0–0)	0 (0–0)	0 (0–0)	0 (0–0)	0 (0–0)	0 (0–2)	0 (0–0)	0 (0–0)	1 (0–3)
	Yes	0 (0–0)	0 (0–0)^a^	0 (0–0)	0 (0–1)	0 (0–0)	0 (0–0)	0 (0–0)	1 (0–2.5)	0 (0–3)^a^	0 (0–0)	2 (0–9)
Pharyngeal residue	No	0 (0–0)	0 (0–0)	0 (0–0)	0 (0–0)	0 (0–0)	0 (0–0)	0 (0–0)	0 (0–1)	0 (0–2)	0 (0–0)	0 (0–4.5)
	Yes	0 (0–0)	0 (0–0)	0 (0–0)	0 (0–0)	0 (0–0)	0 (0–0)	0 (0–0)	1 (0–2)^a^	0 (0–1)	0 (0–0)	2 (0–5.75)
Oropharyngeal dysphagia	No	0 (0–0)	0 (0–0)	0 (0–0)	0 (0–0)	0 (0–0)	0 (0–0)	0 (0–0)	0 (0–0)	0 (0–0)	0 (0–0)	0 (0–3)
	Yes	0 (0–0)	0 (0–0)	0 (0–0)	0 (0–0)	0 (0–0)	0 (0–0)	0 (0–0)	1 (0–2)^b^	0 (0–2)	0 (0–0)	2 (0–6)^b^

Values are presented as medians (interquartile range). PR, pharyngeal residue.

Mann–Whitney test.

a *p* < 0.05.

b *p* < 0.01.

**Fig. 3:**
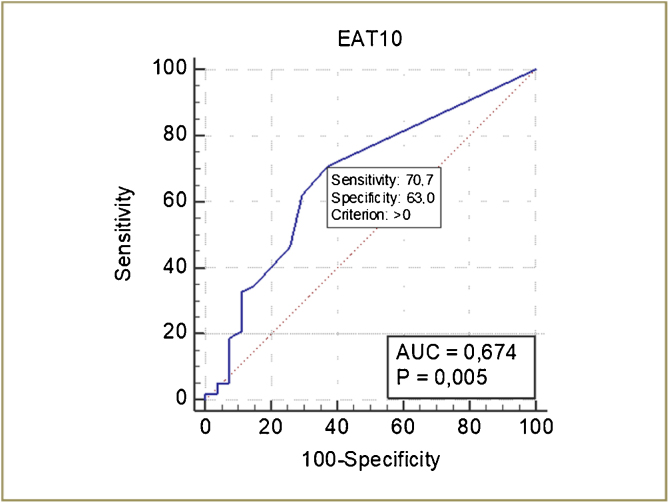
ROC curve of the EAT-10 for detection of oropharyngeal dysphagia.

The ROC curve of the EAT-10 for the detection of OD is shown in Fig. 3. The cutoff score of the EAT-10 for the detection of OD was ≥ 1, with a sensitivity of 70.7% and specificity of 63%. The discriminatory power of EAT-10 was 67.4% (*p* =  0.005) (Fig. 3).

The sensitivity, specificity, accuracy, predictive values, and likelihood ratios of the EAT-10 are shown in Table 3. The sensitivity and accuracy of the EAT-10 was highest with the cutoff score to ≥1 (Table 3).

**Table 3:** Accuracy of the EAT-10 for screening for oropharyngeal dysphagia.

EAT-10	Cutoff score: ≥1	Cutoff score: ≥2	Cutoff score: ≥3
Sensitivity (95% CI)	70.69% (57.3–81.9)	62.07% (48.4–74.5)	46.55% (33.3–60.1)
Specificity (95% CI)	62.96% (42.4–80.6)	70.37% (49.8–86.2)	74.07% (53.7–88.9)
Accuracy	68.24%	64.71%	55.29%
Predictive value +	80.4% (70.9–87.3)	81.8% (70.9–89.3)	79.4% (65.8–88.5)
Predictive value −	50.0% (37.9–62.1)	46.3% (36.4–56.6)	39.2% (31.7–47.2)
LR+ (95% CI)	1.91 (1.1–3.2)	2.09 (1.1–3.9)	1.80 (0.9–3.6)
LR− (95% CI)	0.47 (0.3‒0.8)	0.54 (0.4‒0.8)	0.72 (0.5–1.0)

LR, likelihood ratio.

## Discussion

This is the first study that investigated the accuracy of the EAT-10 for the identification of the risk of OD assessed by FEES in individuals with OSA. Although the literature shows neurogenic changes in the pharynx of patients with OSA,^4^, ^5^, ^6^, ^7^, ^8^ the population in this study showed signals resulting from changes in not only the pharyngeal phase but also suggestive of changes in the oral phase of swallowing, in line with other studies that used the same method of evaluation in individuals with OSA.^12^, ^13^, ^14^, ^15^ The most prevalent characteristics of swallowing that we found are the onset of the pharyngeal phase of swallowing in pyriform sinuses, premature bolus spillage, residue in the vallecula, piecemeal deglutition, delayed posterior leakage and laryngeal penetration during swallowing. In turn, the main symptoms are food stuck in throats, followed by cough when eating.

Similar to other studies, our study found no significant differences in gender,^12^, ^14^, ^15^, ^17^ age,^12^, ^15^, ^17^ smoking status,^15^, ^17^ body mass index,^12^, ^14^, ^15^, ^17^ neck circumference^12^, ^15^, ^17^ or AHI^12^, ^14^, ^15^, ^17^ among individuals with OSA with and without OD.

We found a high prevalence of premature bolus spillage (67.1%), as have most studies that investigated FEES in individuals with OSA, in which the prevalence varied between 64% and 68.2%.^12^, ^13^, ^14^ A group of researchers who used the same evaluation method found the only dissonant results, with a prevalence of 27.3%;^15^, ^17^ however, the average age of this population was approximately 10 years younger than the population in our study. Although the sensitivity of FEES and videofluoroscopy are similar for the detection of premature spillage,^32^ studies that used videofluoroscopy found a lower prevalence of this alteration in individuals with OSA, i.e., between 20% and 58.8%.^16^, ^18^, ^19^ However, this physiological parameter was defined by the entry of the bolus into the pharynx prior to initiation of pharyngeal swallow by most studies,^11^ and therefore, they were not distinguishing the difference between premature spillage and delayed onset of swallowing, which present distinct causes of swallowing dysfunction and consequently swallowing rehabilitation.^25^ The high prevalence of premature bolus spillage indicated that the population in our study had sensory or motor changes in the oral phase that resulted in poor oral control of the bolus.^25^, ^33^

Bolus location at the initiation of the pharyngeal phase of swallowing has not been a condition evaluated in recent research in individuals with OSA. However, in a videofluoroscopic study, 14% of individuals with OSA exhibited premature bolus leakage to the level of the piriform sinuses,^19^ whereas most of the individuals in our study (70.6%) initiated the pharyngeal phase of swallowing after the bolus reached this region, although this in itself does not characterize dysphagia, since the bolus location at the initiation of the pharyngeal phase of swallowing is variable in healthy individuals. Delayed initiation of the pharyngeal phase of swallowing may be due to decreased pharyngeal sensory or delayed motor activity.^25^ Research has indicated that individuals with OSA have a longer latency time and need a greater volume of water to initiate the pharyngeal phase of swallowing induced by the suprapharynx water injection test,^9^, ^34^ which corroborates the hypothesis that the delayed initiation of the pharyngeal phase of swallowing in this population is due to sensory alterations in the pharynx.

Almost half of the participants in this study exhibited piecemeal deglutition and delayed posterior leakage (47.1%), manifestations associated with changes in the oral phase of swallowing in elderly individuals.^28^ Piecemeal deglutition was observed in 28% of individuals with OSA in another study,^14^ while delayed posterior leakage was not a condition evaluated by any of the OSA studies. This sign was due to the escape of the bolus into the pharynx after swallowing due to the changes of the oral phase,^28^ which caused pharyngeal residue.

Thus, the prevalence of pharyngeal residue in our study (61.2%) was slightly higher than that described in the literature, which varies between 22.7% and 55% with FEES^12^, ^13^, ^14^ and between 11.6% and 52.9% with videofluoroscopy.^16^, ^19^ As in another study,^15^ most episodes of residue were located in the vallecula, a signal associated with changes in the ejection force of the tongue,^35^ pharyngeal constriction^36^ or pharyngeal sensory function.^25^

It is known that healthy individuals may exhibit penetration classified as level 2 of the penetration-aspiration scale.^37^ Thus, approximately a third of the individuals in our study exhibited abnormal penetration-aspiration scores (level ≥3 of the penetration-aspiration scale). Our results are in line with other studies that showed that the prevalence of aspiration is very low^14^ or zero^12^, ^13^, ^15^, ^17^, ^18^, ^19^, ^38^ in individuals with OSA. Regarding penetration, our results are similar to those found by Schindler et al.^14^ However, the results presented in the literature are very variable, between 0% and 36%.^12^, ^13^, ^14^, ^15^, ^17^, ^18^, ^19^, ^38^ This variability may be due to the temporal resolution and exam evaluation methods. This is because exams with low temporal resolution may not show the frame in which the patient exhibited penetration. In addition, the evaluation of the frame-by-frame examination allowed us to observe penetration episodes that may not be easily seen in real time.

When penetration-aspiration occurred, it was not identified by any of these surveys. Most episodes of penetration-aspiration in this study population occurred during pharyngeal phase of swallowing, a situation resulting from the delay or deficiency of laryngeal closure mechanisms.^25^

Although the literature has revealed sensory and histological changes in nerves and muscle fibers of the soft palate in individuals with OSA,^7^ this study, as well as others,^12^, ^17^, ^21^ did not show velopharyngeal insufficiency.

The prevalence of OD in our study was the same as that evidenced by another group (68.2%).^12^ According to the literature, the prevalence of OD in the population with OSA ranges from 16% to 78%;^11^ however, the prevalence of OD when diagnosed by FEES varies between 27.3% and 68.2%.^12^, ^14^, ^15^, ^17^ Although a study has shown that the treatment of OSA with CPAP was able to reverse the endoscopic findings of swallowing dysfunction,^15^ our results found no difference in the prevalence of OD between individuals who used and who did not use CPAP. It is noteworthy that all individuals who used CPAP had been using it for more than a year. A possible explanation for this finding refers to the possibility that individuals with OSA have oro-facial myofunctional impairments and craniofacial changes that may impact the swallowing dynamics^18^ and which are not treated by CPAP. According to researchers, individuals with OSA have oromyofunctional impairments, lower hyoid position and reduced time of sustaining the hyoid elevation during swallowing (suggestive of suprahyoid muscles fatigue), which in addition to predispose to upper airway obstruction during sleep might influence swallowing dynamics, including with an impact on the safety of swallowing.^18^ Thus, although CPAP acts on the possible mechanisms responsible for neural injuries in the pharynx, which are identified as causes of swallowing dysfunction in this population, it does not promote adequacy of the oromyofunctional and suprahyoid muscles, which are also identified as possible causes of swallowing dysfunction in these subjects.

In line with the literature, the most prevalent symptom in this population was “food sticks in throat”.^12^ The variation in the total EAT-10 scores in this study was the same as that found by other researchers in individuals with OSA.^39^ The average EAT-10 score in studies evaluating individuals with OSA ranged between 0.37^40^ and 2.86;^39^ however, none of these studies used an associated method of instrumental assessment of swallowing, such as videofluoroscopy or FEES.

Our results showed that the greater the OD severity was, the higher the EAT-10 scores. We already expected that the score of some items on the EAT-10 would not be related to the swallowing changes in individuals with OSA, such as the items “my swallowing problem has caused me to lose weight”, “swallowing is painful” and “swallowing is stressful”. This is because OSA is a predictor of obesity^2^; individuals with OSA have no reason for odynophagia; and manifestations of OD are subclinical in this population,^14^, ^16^, ^19^, ^21^ which in theory would not cause stress during swallowing. Thus, we also expected that the score of the item “the pleasure of eating is affected by my swallowing” was not related to the characteristics of swallowing of these individuals, although we found a relationship with premature bolus spillage. Delayed posterior leakage and residue result in bolus remaining in pharyngeal recesses after swallowing. Thus, it is understandable because the score of the item “when I swallow food sticks in my throat” showed a relationship with delayed posterior leakage, residue and OD.

A result that drew attention was the fact that the score on the items “swallowing liquids takes extra effort” and “swallowing solids takes extra effort” does not show any relationship with any of the signs of swallowing dysfunction, although the FEES showed alterations in swallowing liquids and solids. On the other hand, the score of the item “swallowing pills takes extra effort” showed a relationship with delayed posterior leakage, a signal indicating changes of the oral phase.^28^ A possible explanation for these results may be related to the better perception of difficulty with pills, which requires greater coordination for the organization and ejection of the pill with the food/liquid that helps its swallowing, in addition to providing, in case of difficulty in transporting the bolus, more discomfort. Another possible explanation is related to the excess of pills taken by these individuals, who consumed 10 or more pills a day, often generating an aversion to swallowing pills accompanied by complaints that the large number of pills causes difficulty in swallowing them.

Another expected result was the relationship between the score of the item “I cough when I eat” and penetration-aspiration, since penetration-aspiration can cause coughing to expel bolus from the airways. Although we did not expect a relationship to the score on the item “my swallowing problem interferes with my ability to go out for meals”, it was related to the swallowing changes of this population, due to these being changes subclinical;^14^, ^16^, ^19^, ^21^ it makes sense that individuals who experience coughing and choking while eating may feel embarrassed to eat outside the home.

Finally, it is noteworthy that although the total score on the EAT-10 was higher in individuals with OD compared to individuals without OD, the score in the group with OD was lower than the cutoff score for the risk of dysphagia proposed by the study of cross-cultural adaptation of the EAT-10 to Brazilian Portuguese.^23^ According to the authors, the Brazilian version of the EAT-10 showed a discriminatory power of 72.97%, with a sensitivity of 69.7% and specificity of 72% for the cutoff score ≥3, although the validation process in the Brazilian sample has not yet been fully completed.^23^ The low cutoff score in our study can be explained by the fact that OD was subclinical in this population,^14^, ^16^, ^19^, ^21^ that is, usually without symptoms, and some items of the EAT-10 are not related to the swallowing characteristics in this population. Therefore, future studies should validate a screening instrument for the risk of dysphagia specific to this population.

This study has some limitations. The first was related to the white-out at FEES, which does not allow the identification of penetration-aspiration that occurs during this phase without resulting in residue in the airways although it lasts less than 0.5 s.^32^ The second was related to the sample selection. Despite the exclusion of individuals over 65 years of age due to the physiological changes in swallowing caused by aging, the average age of the population in this study remained advanced, which may have contributed to the high prevalence of some manifestations. In addition, as well as most studies that investigated OD in the population with OSA, we did not evaluate the presence or absence of gastroesophageal reflux, a condition prevalent in individuals with OSA and wich may justify some signs and symptoms of swallowing dysfunction found in this study.^11^ Similarly, we did not evaluate the presence or absence of endolaryngeal findings and symptoms suggestive of laryngopharyngeal reflux or the prevalence of this condition in our sample. The third was the absence of a control group without OSA. The last limitation was that it was a convenience sample, and its size does not allow generalization of the results, although the final sample of this study is larger than that of other studies about dysphagia in patients with OSA.

In conclusion, the population of this study has a high prevalence of dysphagia with changes in the pharyngeal phase of swallowing detected in FEES and changes in the oral phase of swallowing inferred in FEES. In addition, the greater severity of dysphagia was associated with higher EAT-10 scores. The association between EAT-10 and FEES was especially high with signs of delayed posterior leakage, posterior bolus spillage, penetration-aspiration, and pharyngeal residue. In this population of resistant hypertensive patients with OSA, the cutoff score for EAT-10 for screening for OD was ≥1 with a sensitivity of 71% and a specificity of 63%.
